# The Atlanta Freak

**Published:** 1896-11

**Authors:** 


THE ATLANTA FREAK: The vacuous young egotist (self-styled "Ralcy,") who conducts the obscene department of a literary freak at Atlanta, Ga., calls the TEXAS MEDICAL JOURNAL "a wild-eyed, red-back Texan steer." Goodness gracious! And we hadn't *done a thing* to him, either. (See our September number). "Texan steer" is good; it is as if we should say "a Georgi*an* idiot," for instance. (He must have had blood in his own eye, as the *Southern Medical Record*, of Atlanta, whom the young chap had, with the characteristic, rashness and impetuosity of callow youth, indiscreetly attacked—speaks of him as "Ralcy *red-i*-divus.") It is right funny to see offenders of that class *squirm* when the "Red Back" gets after them.

But "Ralcy" struck a hornets' nest, so to speak, when he criticised a paragraph in the *Southern Medical Record* as being "indecent." (Kettle and pot). The editor of the *Record* literally excoriates the young man and the entire establishment,—"*Moody's Medical Magazine*," speaking of the young "editor" as one "recently regurgitated from the State Medical Association for gross violation of the Code of Ethics," and of the proprietors of the nondescript publication as "slick gentry from the north," who are "evidently working certain respectable medical gentlemen to the queen's taste," or words to that effect. *Regurgitated!*—i. e., spewed out, puked up and spit out, utterly rejected as unwholesome and disagreeable! My!

The *Record* very justly criticises a policy which leads to the absurd attempt to mix medical and polite literature, and supposes, for instance, that in accordance with that "policy," (of which, by the bye, the publishers claim, in their prospectus, to "have a clear idea")—there should appear in the same issue—a lovely editorial, say, on the Beautiful Snow, or on Bobbie Burns and the little mouse (see No. 3), side by side with an illustrated paper on the operative treatment of piles! What a delectable dish for the family fireside! *M. M. M.* "em"braces too much; it is a monstrosity, and will die in the bornin'.

